# Supplementary figures and images for: Reevaluation of Piezo1 as a gut RNA sensor
Source: eLife. 2022 Nov 16;11:e83346. doi: 10.7554/eLife.83346 (PMC9691018; doi:10.7554/eLife.83346)

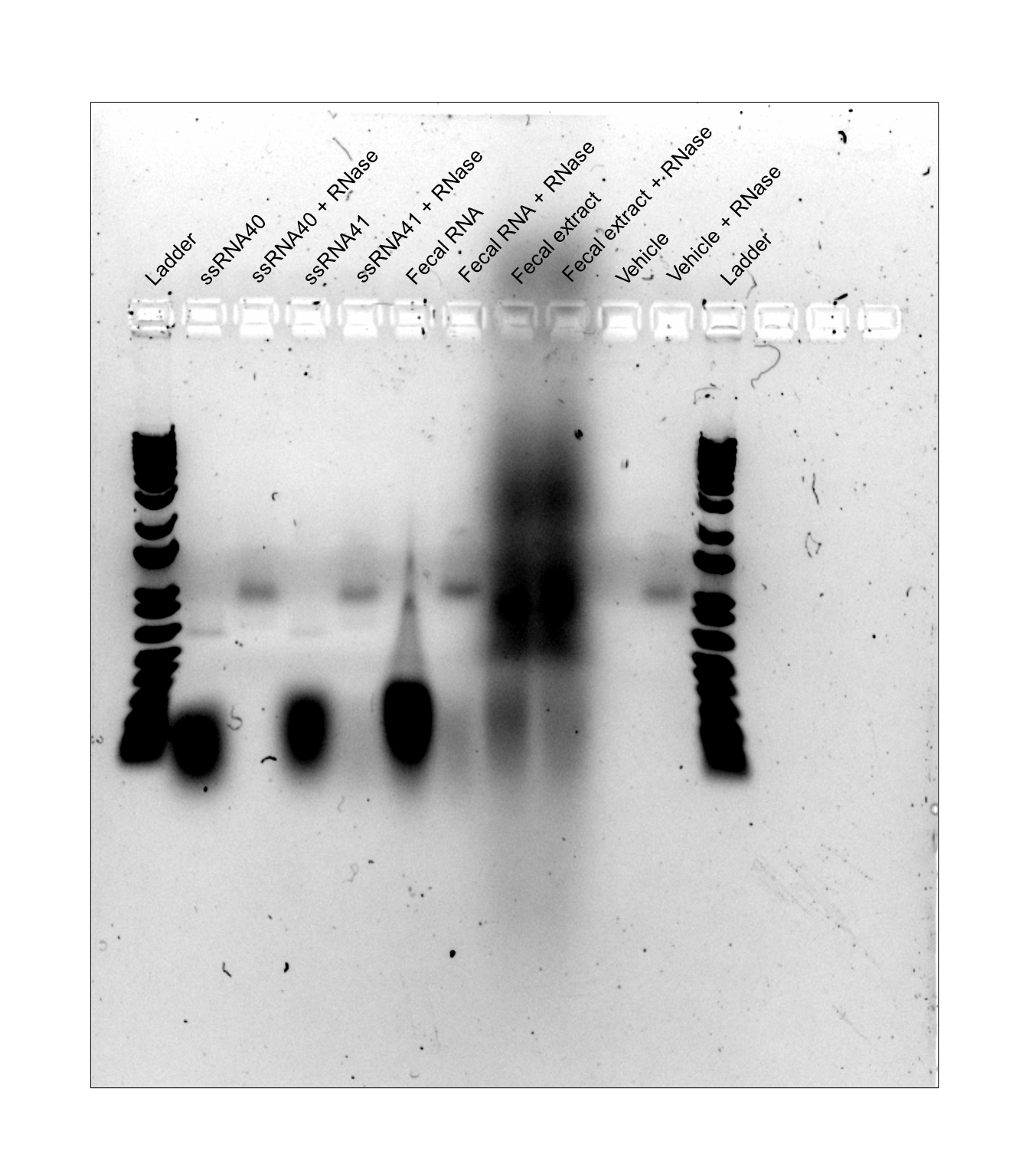

Supplement: Figure 3—source data 1. [file elife-83346-fig3-data1.zip › Figure 3-source data 1/Figure 3 source data.tif]

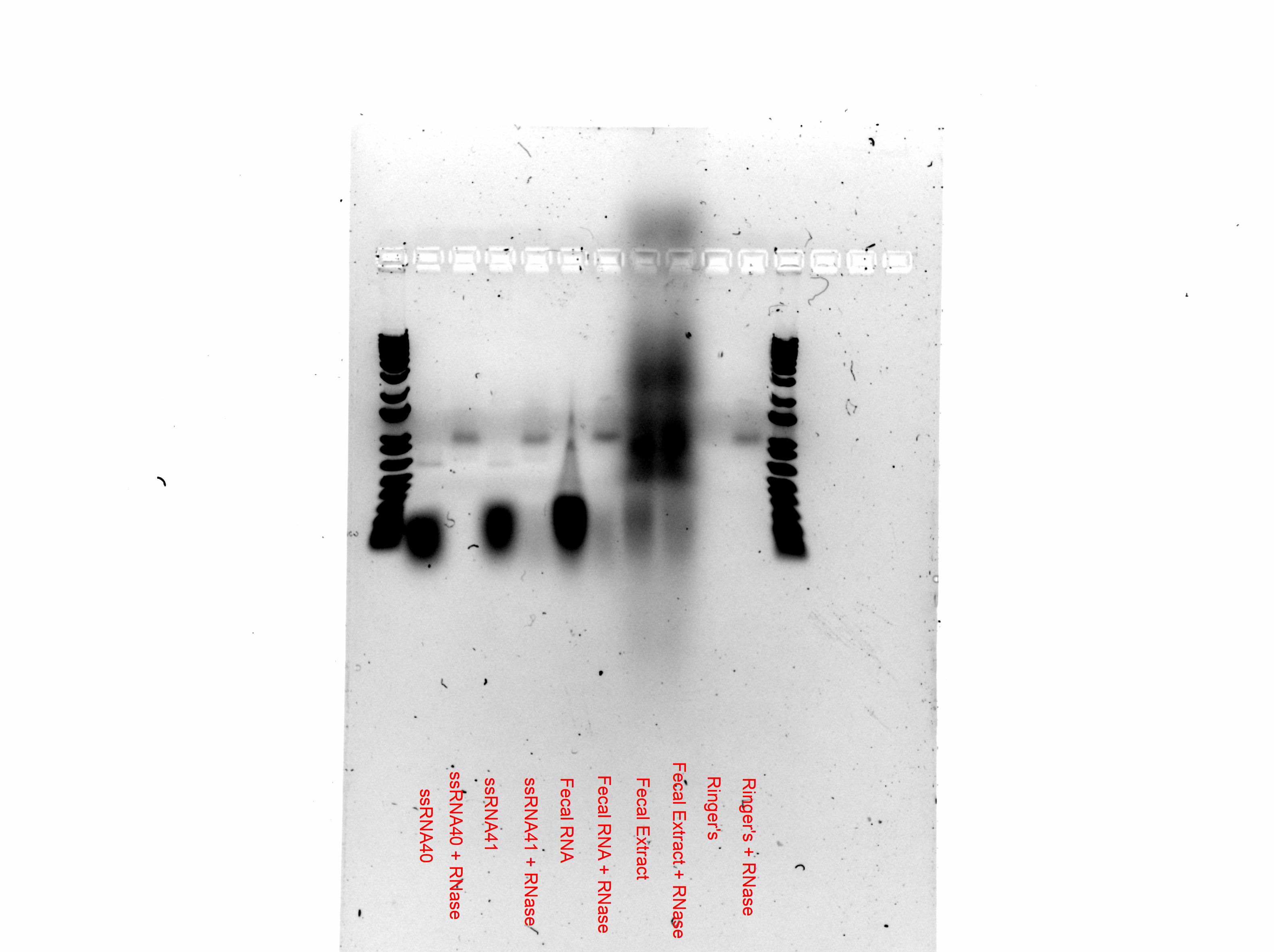

Supplement: Figure 3—source data 1. [file elife-83346-fig3-data1.zip › Figure 3-source data 1/Original gel file.tif]
